# The demineralization resistance and mechanical assessments of different bioactive restorative materials for primary and permanent teeth: an in vitro study

**DOI:** 10.1038/s41405-024-00209-4

**Published:** 2024-04-05

**Authors:** Maria Salem Ibrahim, Fahad Rakad Aldhafeeri, Abdullah Sami Banaemah, Mana S. Alhaider, Yousif A. Al-Dulaijan, Abdulrahman A. Balhaddad

**Affiliations:** 1 Department of Preventive Dental Sciences, College of Dentistry, Imam Abdulrahman Bin Faisal University, P.O. Box 1982, 34212 Dammam, Saudi Arabia; 2 College of Dentistry, Imam Abdulrahman Bin Faisal University, P.O. Box 1982, 31441 Dammam, Saudi Arabia; 3 Department of Substitutive Dental Sciences, College of Dentistry, Imam Abdulrahman Bin Faisal University, P.O. Box 1982, 31441 Dammam, Saudi Arabia; 4 Department of Restorative Dental Sciences, College of Dentistry, Imam Abdulrahman Bin Faisal University, P.O. Box 1982, 31441 Dammam, Saudi Arabia

**Keywords:** Bonded restorations, Composite resin

## Abstract

**Objectives:**

This article examines the efficacy of two bioactive dental composites in preventing demineralization while preserving their mechanical and physical properties.

**Materials and methods:**

The study compares Beautifil Kids and Predicta® Bioactive Bulk-Fill (Predicta) composites with conventional dental composite. Flexural strength and elastic modulus were evaluated using a universal testing machine. A pH-cycling model assessed the composites’ ability to prevent dentin demineralization. Color stability and surface roughness were measured using a spectrophotometer and non-contact profilometer, respectively, before and after pH-cycling, brushing simulation, and thermocycling aging.

**Results:**

Beautifil Kids exhibited the highest flexural strength and elastic modulus among the materials (*p* < 0.05). Predicta demonstrated the highest increase in dentin surface microhardness following the pH-cycling model (*p* < 0.05). All groups showed clinically significant color changes after pH-cycling, with no significant differences between them (*p* > 0.05). Predicta exhibited greater color change after brushing and increased surface roughness after thermocycling aging (*p* < 0.05). While Beautifil Kids had higher surface roughness after pH-cycling (*p* < 0.05).

**Discussion/Conclusion:**

Bioactive restorative materials with ion-releasing properties demonstrate excellent resistance to demineralization while maintaining mechanical and physical properties comparable to the control group.

## Introduction

Dental caries is a prevalent oral health issue that affects the teeth and causes localized tooth structure destruction [1]. Despite the methods used to prevent it, it is still widely prevalent, representing one of the public health challenges in dentistry [2]. Dental caries is a highly complicated biological mechanism in its origin due to a persistent divergence between the mineral levels of teeth and the fluids present in biofilms, leading to a state of physiological imbalance [3]. Caries’ risk factors include many aspects, most importantly, low fluoride intake, existing in a low-income society, and inadequate oral hygiene [4, 5]. Early loss of minerals (demineralization) from the tooth structure is characterized by the change of color and texture of the tooth. When no proper intervention using remineralization tools is applied, the continuous loss of minerals can lead to physical tooth destruction, where restoring the defective tooth structure after caries ablation is the treatment choice [6].

Unfortunately, teeth with dental restorations are more susceptible to carious lesions than intact teeth. In particular, resin composites accumulate plaque more than other restorative materials such as amalgam and glass ionomer [7, 8]. However, resin composites are still the most used restorative materials due to their highly esthetic appearance despite being more likely to be affected by secondary caries [9, 10]. To improve the resistance against cariogenic bacterial species of resin-based materials, different approaches were suggested to impart bioactivity and antibacterial properties into resin-based composite formulations [9, 10].

To accommodate the downsides of conventional restorative materials and improve their resistance against secondary caries, studies have investigated new bioactive resin-based materials that might aid in reversing the demineralized tooth structure by favoring the remineralization process via the release of ions [5, 11]. In particular, the combination of fluoride (F^−^) with other ions such as calcium (Ca^2+^) and phosphate (P_4_^3−^) released from restorative materials seems to be beneficial in terms of caries reduction [12]. Several investigations advocate that the long-term release of Ca^2+^ and P_4_^3−^ ions from resin-based dental materials has valuable impacts [13, 14]. These bioactive materials can play a role in halting the advancement of caries and enabling infected tissues to undergo repair. These materials possess the ability to release, absorb, and subsequently re-release essential ions such as Ca^2+^, P_4_^3−^, and F^−^. These ions act as a reservoir, working to counteract the demineralization development once it initiates [15–17].

Besides being bioactive, these resin composite formulations must reveal excellent mechanical and physical properties to maintain their integrity and surface characteristics when subjected to the force of chewing and other physiological challenges inside the oral cavity [6, 10, 18]. A material with good antibacterial properties is useless if its strength is reduced over time. Several investigations demonstrated that several bioactive resin composite formulations may exhibit decay in their mechanical and physical features following different aging conditions, which may cause these formulations fail mechanically [6, 10, 18].

With the revolution of bioactive resin composites, evaluating the mechanical properties, color stability, and surface characteristics is essential to ensure excellent clinical service. Only a few studies have investigated the performance of newly commercially available bioactive resin-based restorative materials [16, 19–23], highlighting the need for further evaluation from different perspectives. As a result, this laboratory study aims to assess the potential of different bioactive restorative materials to inhibit demineralization and promote remineralization, and their mechanical, physical, and biological performance following different challenging conditions.

## Methodology

### Ethical approval and study design

This research is a laboratory study conducted in a blinded and randomized manner. The study received approval from the Institutional Review Board (IRB 2021-02-484) at Imam Abdulrahman Bin Faisal University. The study investigated the mechanical properties, physical characteristics, and demineralization resistance of three restorative materials (Table 1). The mechanical properties were assessed via flexural strength and elastic modulus. The demineralization resistance was evaluated by measuring the microhardness of tooth dentin filled with the three restorative materials. The physical characteristics were evaluated by recording the change in color and surface roughness before and after three challenges: pH-cycling, brushing simulation, and thermocycling aging.

**Table 1:** The restorative materials used in this study (type, chemical composition, and manufacturer).

Name	Type	Composition	Manufacturer
Blank	Negative control	–	–
G-ænial^TM^ Universal Flo. Composite	Non-bioactive Universal Light-cured Flowable Composite Material	- Matrix (31%): Urethanedimethacrylate, Bis-MEPP, TEGDMA - Filler (69% wt., 50% vol.): Silicon dioxide 16 nm, Strontium glass 200 nm, Pigment - Initiator (Trace): Photo initiator	GC America, Chicago, IL USA.
Beautifil Kids SA	Bioactive Light-cured Self-Adhesive Flowable Restorative	- Silicon dioxide, Zirconium silicate, Glass powder, UDMA (10–30%), 2-HEMA (5–10%)	SHOFU, Kyoto, Japan
Predicta® Bioactive Bulk-Fill (Predicta)	Bioactive Dual-Cure Flowable Low Viscosity Bulk-Fill Restorative	- Poly(oxy-1,2-ethanediyl), .alpha.,.alpha.ˈ-[(1-methylethylidene)di-4,1-phenylene]bis[.omega.-[(2-methyl-1-oxo-2-propenyl)oxy]- (10–20%) - 2-Propenoic acid, 2-methyl-, 1,6-hexanediyl ester (4.2–11%) - 2-Propenoic acid, 2-methyl-, (1-methylethylidene)bis[4,1-phenyleneoxy(2-hydroxy-3,1-propanediyl)] ester (0.8–4%) - Dibenzoyl peroxide (0.0097–0.97%)	Parkell, Edgewood NY, USA

### Flexural strength and elastic modulus

Samples (*n* = 6) for flexural strength and elastic modulus testing were fabricated in stainless steel mold (2 mm × 2 mm × 25 mm) following the ISO 4049 protocol [24]. The material was inserted into that mold. Glass slaps and polyester strips were placed below and on the top of the mold. The samples underwent a 20-s light-curing process from both sides (Satelec Mini LED Curing Light 1250 mW/cm^2^, A-dec Inc., Newberg, OR, USA), and then placed in 37 °C incubator for 24 h. Flexural strength and elastic modulus were measured using a 10-mm span in a universal testing machine (INSTRON 5965 load frame, Boston, MA, USA) at a crosshead-speed of 1 mm/min [25]. The following formulas were used:

1 $${{F}}=(3{{LS}})/({2{{WH}}}^{2})$$

whereby *F* = flexural strength, *L* = maximum load, *S* = span, *W* = width of the specimen and *H* = height.

2 $${{M}}=({{{LS}}}^{3}3)/({4{{WH}}}^{3}3{{d}})$$

whereby *M* = elastic modulus, *L* = maximum load, *S* = span, *W* = width of the specimen, *H* = height and *d* = defluxion corresponding to the load (L).

### pH-cycling model

#### Sample preparation

Human teeth that were recently extracted were gathered and cleansed using ethanol. The intact dentin portions of the teeth were carefully separated from the crown using a separating disk and subsequently affixed to a cylindrical acrylic block. The acrylic base was leveled, after which the dentin side was sequentially polished using a polishing machine (BUEHLER MetaServ 250 Grinder–Polisher with Vector Power Head, Hong Kong, China) with the aid of silicon carbide sandpapers of 320-Grit, 500-Grit, and 1200-Grit, while water was used as a lubricant during the process [26, 27]. The initial surface microhardness was assessed utilizing a microhardness tester (BUEHLER MicroMet 6040 Hardness Tester, Shanghai, China). The measurements were obtained by averaging five indentations spaced 100 µm apart, created using a diamond Knoop head with a 25-g force applied for a duration of 10 s [26, 27]. Teeth samples exhibiting an average microhardness of 75 KNH ± 20% were selected for inclusion in the study. These prepared teeth samples were then distributed blindly and randomly across the three materials used in the study: G-ænial^TM^ Universal Flo. Composite. Composite, Beautifil Kids SA, and Predicta® Bioactive Bulk-Fill (Predicta). A fourth blank group with no restorations was used to serve as the negative control. Each material was applied to the dentin surface and subsequently light-cured for a duration of 20 s using a light-emitting source. (Satelec Mini LED Curing Light 1250 mW/cm^2^, A-dec Inc., Newberg, OR, USA).

#### pH-cycling protocol

A 7-day pH-cycling model of demineralization and remineralization cycles was used [28]. The samples were placed in a demineralization solution, with each sample immersed in 30 ml of the solution: 2.0 mmol/l calcium (Ca(NO_3_)2·4H_2_O), 2.0 mmol/l phosphate (NaH_2_PO_4_·2H_2_O), 0.075 mmol/l acetate buffer and 0.02 ppm F at pH 4 for 6 h at room temperature. Subsequently, the samples were subjected to a thorough rinse with distilled water, followed by drying, and then submerged in a remineralization solution, with each sample immersed in 15 ml of the solution: 1.5 mmol/l calcium (Ca(NO_3_)2·4H_2_O), 0.9 mmol/l phosphate (NaH_2_PO_4_·2H_2_O), 150 mmol/l potassium chloride, 0.03 ppm fluoride standard, and 0.1 mol/l tris buffer at pH 7 at room temperature for 18 h. This cycling protocol was repeated for 7 days [28]. The restorative materials were gently detached from the dentin surface, and the following assays were conducted.

#### Surface hardness change

The post-intervention microhardness measurements, after the pH-cycling model (*n* = 4), were obtained utilizing the identical parameters employed during the baseline measurements described in the “Sample preparation” section.

#### Color change

To assess color change, a reflectance spectrophotometer (Color-Eye 7000A; X-rite, Grand Rapids, MI, USA) was utilized. The Commission Internationale de l’Elcairage (CIE) L*a*b*color scale was employed to quantity the color of the test samples before and after the pH-cycling model. The CIE Lab* system constitutes a three-dimensional color space, where L* represents lightness on a scale of zero to 100, ranging from black to white. On the other hand, a* and b* represent chromaticity without specific numerical boundaries. Negative a* values indicate green shades, positive a* values indicate red shades, negative b* values correspond to blue shades, and positive b* values correspond to yellow shades [26]. The three color coordinates were documented at the two distinct time intervals, and ΔE* values were computed by employing the formula to measure the color difference before and after the pH-cycling model [26]:

$${\Delta {{E}}}^{* }={[{({\Delta {{L}}}^{* })}^{2}+{({\Delta {{a}}}^{* })}^{2}+{({\Delta {{b}}}^{* })}^{2}]}^{1/2}$$

#### Surface roughness change

Surface roughness was assessed employing a high-resolution non-contact optical profiler (Contour GT-K 3D Optical Microscope, Bruker, Billerica, MA, USA) in a three-dimensional manner. The samples were scanned at three distinct areas, and the arithmetical mean of the roughness profile (Ra) was obtained [29]. For the analysis and graphical representation, the Vision 64 software (Bruker, Billerica, MA, USA) was utilized, and the average roughness value was calculated based on three areas for each sample [29]. The measurements were done at the baseline and after the pH-cycling was completed.

### Brushing simulation and the evaluation of color change and surface roughness

Circular disks (10 mm × 2 mm) samples (*n* = 5) were fabricated using a circular mold. The samples underwent a 20-s light-curing process from both sides (Satelec Mini LED Curing Light 1250 mW/cm^2^, A-dec Inc., Newberg, OR, USA). Medium-bristled toothbrushes were positioned over the occlusal surface of the specimens. Utilizing a brushing simulation device (Model ZM-3.8, SD Mechatronik, Feldkirchen-Westerham, Germany), the toothbrushes were adapted to brush in a mesiodistal path. A constant force of 280 g of pressure and 15,000 strokes were applied, equivalent to 20 strokes per day over a span of 2 years. Samples’ color parameters were recorded as in the “Color change” section. Surface roughness before and after the brushing simulation was recorded as in the “Surface roughness change” section. This was measured before and after the brushing protocol was completed.

### Thermo-cycling model and the evaluation of color change and surface roughness

Circular disks (10 mm × 2 mm) samples (*n* = 5) were fabricated as described in the previous section. The prepared samples (*n* = 5 per group) of all groups were put in thermocycling (Thermocycler THE-1100—SD Mechatronik GmbH, Feldkirchen-Westerham, Germany) between 5° and 55° for 5000 cycles to act out 6 months in the mouth [30]. Samples’ color parameters were recorded as in the “Color change” section. Surface roughness before and after thermos-cycling was recorded as in the “Surface roughness change” section. This was measured before and after the thermos-cycling was completed.

### Statistical analysis

To assess data normality, the Shapiro–Wilk test was employed. The results were analyzed using one-way analysis of variance (ANOVA) and Two-sample *t*-test. Bonferroni’s multiple comparison tests were utilized for conducting multiple comparisons between the groups under study. All statistical analyses were carried out using Stata/IC 14.2 software (Stata, College Station, TX, USA) with a significance level set at alpha = 0.05.

## Results

**Fig. 1:**
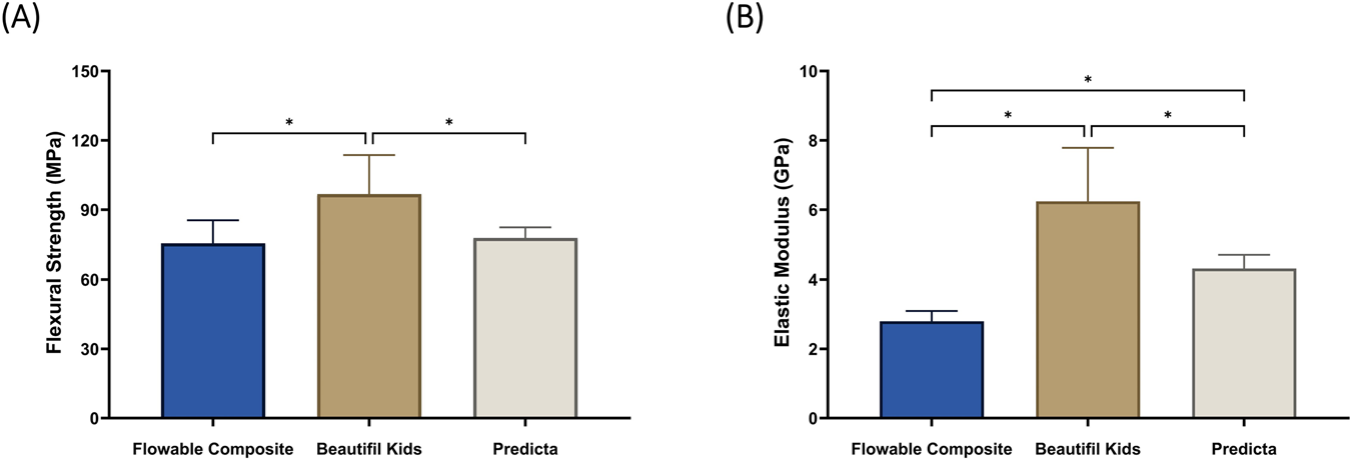
The mechanical assessment of the materials. The flexural strength (**A**) and elastic modulus (**B**) of the three restorative materials (mean ± SD). Stars denote statistically significant difference between the groups (*p* < 0.05).

Figure 1 and Table 2 represent the flexural strength (Fig. 1A) and elastic modulus (Fig. 1B) of the studied materials. Beautifil Kids presented the highest flexural strength (96.74 ± 16.88) and elastic modulus (6.24 ± 1.55), which were statistically significant from the other groups (*p* < 0.05).

**Table 2:** Means and standard deviations of flexural strength and elastic modulus of the studied materials.

Material Property	Flowable composite	Beautifil Kids	Predicta
Flexural strength (Mean ± SD in MPa)	75.68 ± 9.74^a^	96.74 ± 16.88^b^	77.97 ± 4.43^a^
Elastic modulus (Mean ± SD in GPa)	2.79 ± 0.30^a^	6.24 ± 1.55^b^	4.32 ± 0.40^c^

Different superscript letters denote statistically significant difference between the groups (*p* < 0.05).

**Fig. 2:**
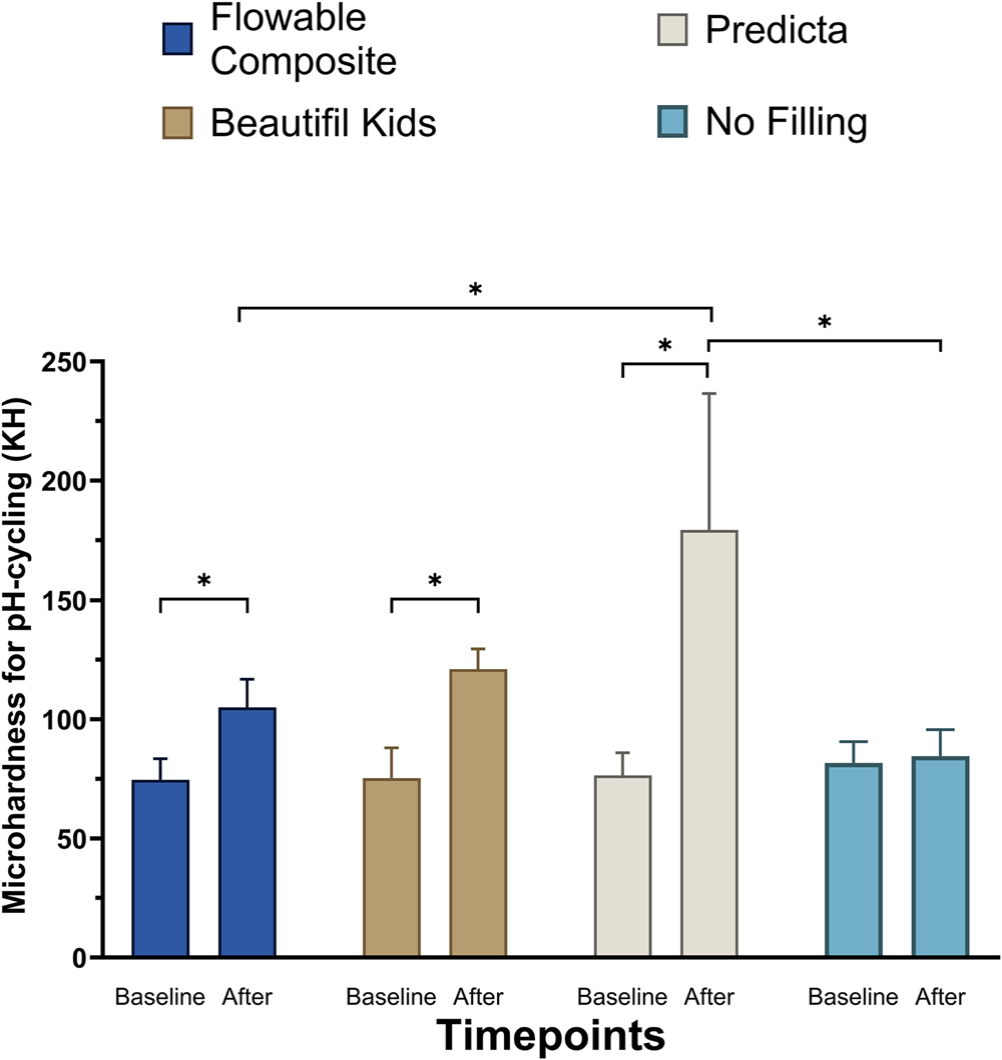
The demineralization restorative of the restorative materials. Dentin surface microhardness after the pH-cycling. Stars denote statistically significant difference between the groups (*p* < 0.05).

Figure 2 and Table 3 summarize the changes in dentin surface microhardness after the pH-cycling protocol was completed. Predicta group showed an increase in dentin surface microhardness. This increase was significantly (*p* < 0.05) higher than the other two materials and the control group (dentin sample with no restoration). The dentin samples without any restorative material did not show any significant changes after completing the pH-cycling protocol. However, dentin surface microhardness under Beautifil Kids and flowable composites increased after the pH-cycling protocol was completed.

**Table 3:** Means and standard deviations of dentin microhardness under each of the studied materials before and after pH-cycling.

Material Property	Flowable composite	Beautifil Kids	Predicta	No filling
Microhardness before pH-cycling (KH)	74.63 ± 8.84^aA^	75.3 ± 12.67^aA^	76.48 ± 9.41^aA^	81.63 ± 9.0^aA^
Microhardness after pH-cycling (KH)	105.0 ± 11.89^aB^	121.0 ± 8.43^abB^	179.3 ± 57.28^bB^	84.57 ± 11.13^aA^

Different superscript small letters denote statistically significant difference between the groups and different superscript capital letters denote statistically significant difference between the two time = points (before and after) within the same material group (*p* < 0.05).

**Fig. 3:**
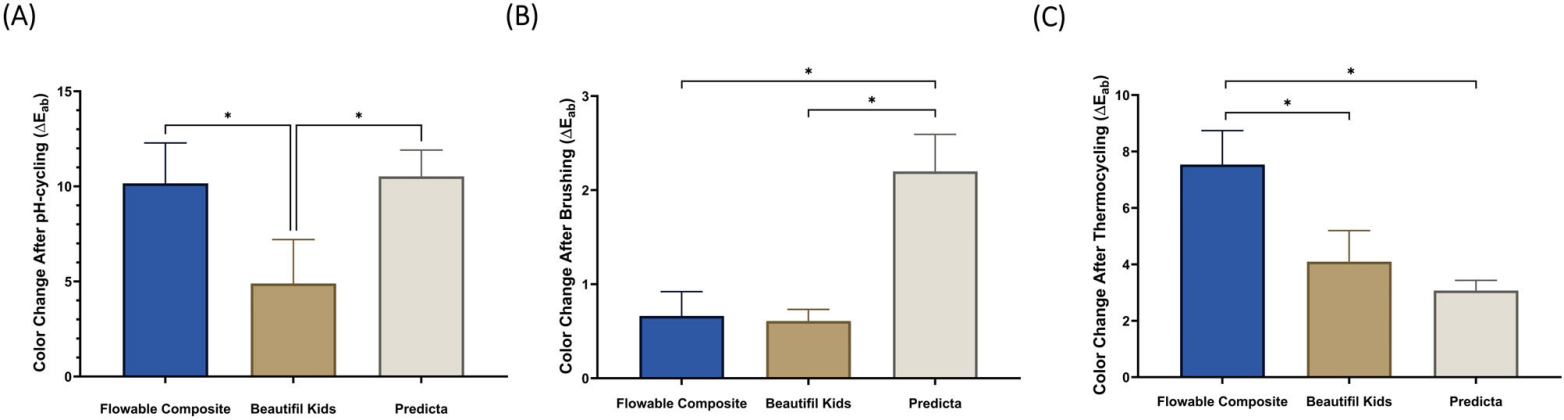
The color stability of the restorative materials. Color change (∆*E*_ab_) values (mean ± SD) of the studied groups after pH-cycling (**A**), brushing simulation (**B**), and thermocycling (**C**). Stars denote statistically significant difference between the groups (*p* < 0.05).

Figure 3 and Table 4 demonstrate the means and standard deviation of color changes (Δ*E*_ab_) following the three different aging protocols: pH-cycling (Fig. 3A), brushing (Fig. 3B), and thermocycling (Fig. 3C). After samples were exposed to the pH-cycling protocol, all three materials showed changes in color that were clinically significant (above 4). After the brushing protocol was completed, Predicta samples showed the highest change in color in comparison to the other groups (*p* < 0.05). However, the three groups displayed slight changes which were below the clinical noticeable level. After the thermocycling protocol was completed, flowable composite showed the highest change in color (7.5 ± 1.2), followed by Beautifil Kids (4.1 ± 1.1) then Predicta (3.1 ± 0.4).

**Table 4:** Means and standard deviations of color change and surface roughness change of the studied materials after pH-cycling, and brushing, thermocycling.

	pH-cycling			Brushing			Thermocycling		
	Flowable composite	Beautifil Kids	Predicta	Flowable composite	Beautifil Kids	Predicta	Flowable composite	Beautifil Kids	Predicta
Color change (∆*E*_ab_)	10.15 ± 2.13^a^	4.89 ± 2.31^b^	10.52 ± 1.40^a^	0.66 ± 0.26^a^	0.61 ± 0.12^a^	2.20 ± 0.40^b^	7.54 ± 1.20^a^	4.09 ± 1.10^b^	3.07 ± 0.36^b^
Surface roughness (µm or nm)*	−0.76 ± 0.40^a^	0.62 ± 0.45^b^	−1.55 ± 0.43^a^	164.50 ± 73.02^a^	61.30 ± 33.704^a^	122.30 ± 38.96^a^	−85.95 ± 46.13^a^	−25.74 ± 9.83^a^	121.7 ± 67.04^b^

Different superscript letters denote statistically significant difference between the groups (*p* < 0.05).

*: µm in pH-cycling or nm in brushing and thermocycling.

**Fig. 4:**
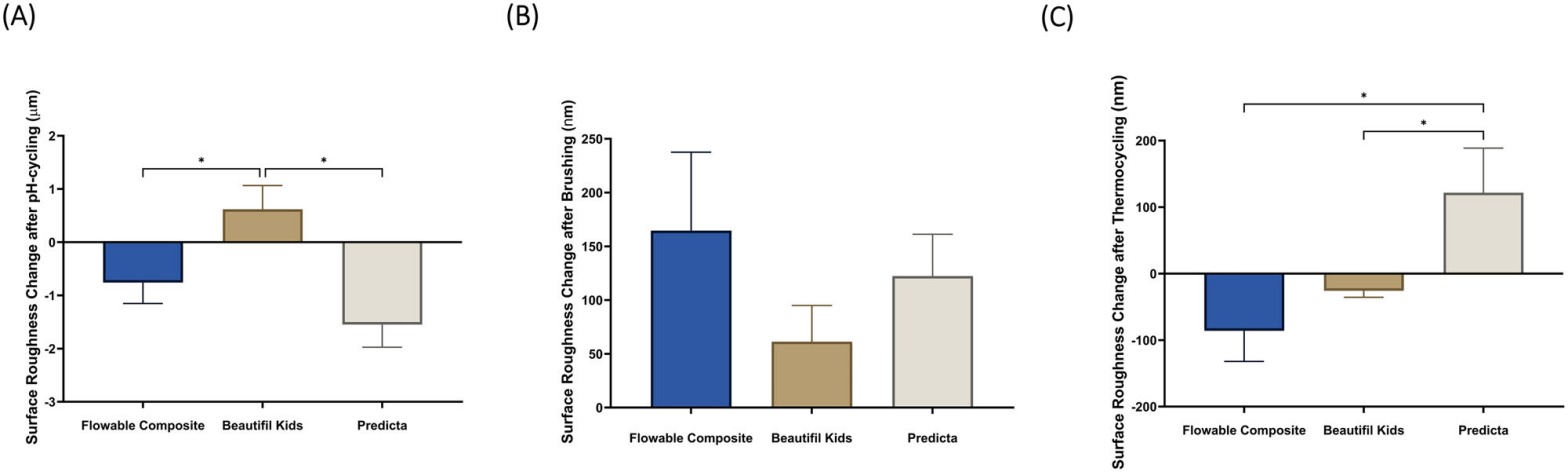
The topographic assessment of the restorative materials. Surface roughness (Ra) in nm (mean ± SD) of the studied groups after pH-cycling (**A**), brushing simulation (**B**), and thermocycling (**C**). Stars denote statistically significant difference between the groups (*p* < 0.05).

Figure 4 and Table 4 demonstrate the means and standard deviation of surface roughness following the three different aging protocols: pH-cycling (Fig. 4A), brushing (Fig. 4B), and thermocycling (Fig. 4C). After samples were exposed to the pH-cycling protocol, Beautifil Kids revealed higher surface roughness compared to the other two materials (*p* < 0.05). After the brushing protocol was completed, surface roughness was amplified in all three groups with no significant differences between the groups (*p* > 0.05). After the thermocycling protocol was completed, flowable composite and Beautifil Kids showed decreases in surface roughness, while Predicta had increased surface roughness and was statistically significant from the other two groups (*p* < 0.05).

## Discussion

Secondary caries is a challenge encountered in restorative dentistry when placing dental restorations, particularly those made of polymeric materials. It refers to the occurrence of new decay around the edges of an existing restoration, which often becomes a site for plaque buildup and biofilm formation [5]. The development of secondary caries follows a similar process as primary caries, involving demineralization, disruption of mineral balance, and enzymatic degradation of tooth structures [6]. However, the presence of a restoration margin can alter this process and contribute to additional tooth damage [6]. Therefore, designing bioactive restorative materials to down-regulate the demineralization process around dental restorations is a priority in restorative dentistry [9, 11]. To fulfill this need, several available brands have been released in the market with ion-releasing or antibacterial capabilities. However, their efficiency in preventing demineralization is not yet explored. Therefore, this study investigated the demineralization protection and the physical properties of two bioactive restorative materials, Beautifil Kids and Predicta.

This study revealed that bioactive restorative materials may protect teeth from demineralization when subjected to severe acidic challenge. This protection was achieved with surface roughness and color changes comparable to the control with no bioactivity. Our results may imply that the bioactive restorative materials tested here, Beautifil Kids and Predicta, can reinforce the tooth structure via the release of remineralizing ions, which can prevent the onset of demineralization at the tooth-restoration interface. This can improve the sustainability of polymeric restorative materials, leading to less restoration replacement and enhanced quality of dental services.

During the lifespan of dental restoration, there are micro-environmental fluctuations at the interface between the tooth and the restoration that can trigger cycles of mineral loss and gain, known as demineralization and remineralization [31, 32]. It is crucial to comprehend the mechanisms underlying these processes to develop advanced strategies for effectively managing the development of both primary and secondary caries. Calcium (Ca^2+^), phosphate (PO_4_^3−^), and fluoride (F^−^) ions play significant roles in these cycles [33, 34]. When these ions are released from a restorative material following a demineralization attack, they can reinforce the tooth structure and prevent mineral loss [33, 34]. For a considerable period of time, fluoride (F) has been recognized for its efficacy in caries prevention through the reduction of tooth dissolution and the enhancement of tooth remineralization processes [35]. Numerous studies have observed that fluoride can directly deposit fluorapatite (FA) or fluoridated hydroxyapatite (FHA) onto the affected tooth surface or facilitate the conversion of other calcium phosphate phases into FA or FHA [33, 34]. Consequently, the formation of FA and FHA can decrease the dissolvability of enamel and dentin. On the other hand, Ca^2+^ and PO_4_^3−^ ions allow the deposition of hydroxyapatite layer, which also can reinforce the strength of the demineralized tooth structure [36].

In this study, the cycles of mineral loss and gain were achieved using a pH-cycling model, where the tooth-restoration interface was subjected to repetitive exposure to remineralization and demineralization conditions. Our results demonstrated that Beautifil Kids and Predicta materials reinforced dentin microhardness compared to their baseline values (Fig. 2). Comparing the brands, Predicta microhardness value after the pH cycling was significantly higher than Beautifil Kids and the controls, while Beautifil Kids value was slightly higher than the control composite. Such findings could be related to the inorganic contents of these two bioactive materials, as Beautifil Kids releases only F^−^ ions, while Predicta releases F^−^, Ca^2+^, and P_4_^3−^ ions. It is expected that the synergetic effects between different remineralizing ions released by Predicta contributed to the significant increase in dentin microhardness.

Our model belongs to the chemical secondary caries model. This model has been adapted in several studies, representing one of many types of in vitro secondary caries models in dentistry, which are chemical, microbial, or combined [37, 38]. In chemical models, either demineralization or a combination of demineralization and remineralization mixtures are used to induce teeth mineral loss, as no microbial planktonic or biofilm growth is involved [37, 38]. In the initial evaluation of bioactive restorative materials and their protection against demineralization in vitro, both chemical and microbial models are useful, especially chemical models where many confounders can be controlled [38, 39]. This assessment is essential to be conducted before testing bioactive restorative materials in situ or in vivo, where the complexity of the oral environment can be experienced [38, 39].

It is critical for any dental composite to maintain its mechanical and physical properties over time to ensure excellent clinical service. There is a specific concern related to the sustainability of bioactive restorative materials, as the release of ions may compromise the integrity of such systems [18]. As a result, a fundamental assessment of the mechanical properties of newly bioactive developed formulations is necessary. It is crucial to recognize that materials exhibiting high bioactivity may experience mechanical failure if their mechanical properties are inadequate, as restorative materials are subjected to repetitive loading and fatigue within the mouth due to the forces of chewing and regular contact with oral fluids and beverages [18]. Our mechanical assessment revealed that the bioactive restorative materials tested here exhibited mechanical performance comparable to the control material with no bioactive ingredients. Future investigations might conduct further analysis related to the long-term assessment of the mechanical properties or testing these materials in dynamic motion.

Among different physical properties, color stability is of significant importance it directly affects the esthetic appearance and longevity of restorations [40, 41]. The color stability of dental composites refers to their ability to maintain their original shade over time, despite exposure to various environmental factors and oral conditions [40, 41]. Dental restorations are subjected to a range of challenges, including exposure to food and beverages, oral hygiene practices, and natural wear and tear. These factors could lead to discoloration, compromising the esthetic outcome of the restoration and reducing patients’ satisfaction [42].

In this study, we conducted three challenges to simulate different conditions inside the oral cavity that might alter the physical characteristics of dental restorations. These conditions included pH cycling, oral hygiene measures (brushing), and thermocycling aging. After subjecting the restorations to pH cycling, all groups exhibited severe color change, with no significant difference between them (Fig. 3A). This suggests that highly acidic solutions have exaggerated effects on the color of dental restorations. After simulating 2 years of brushing, Predicta showed a significant color change compared to Beautifil Kids and the control group (Fig. 3B). However, this finding should be interpreted cautiously for several reasons. Firstly, although there was a statistically significant difference between Predicta and the other groups, the actual color change (Δ*E* value) was around 1.5, which is clinically insignificant. Secondly, all Δ*E* values remained below the critical value of 3. Research has shown that a Δ*E* value higher than 3 is typically noticeable to the naked eye in a clinical setting [43].

On the other hand, following thermocycling aging, Predicta exhibited less color change compared to the other two groups, with the control group showing the maximum changes (Fig. 3C). Overall, the differences in color stability among the tested groups varied depending on the challenges applied. However, it can be generally observed that the color stability of bioactive formulations was comparable to that of the control composite. In summary, the color stability of bioactive formulations was similar to that of the control composite, although some differences were observed depending on the specific challenge. It is important to emphasize the clinical significance of color changes and the overall performance of dental restorations when evaluating their color stability.

When assessing the average surface roughness (Ra) of the tested composites, similar observations were made, with no clear pattern emerging following the three challenging conditions. After pH cycling, Beautifil Kids displayed a substantial difference in surface roughness compared to the other groups (Fig. 4A). However, following thermocycling, Predicta exhibited the highest surface roughness values compared to the other groups (Fig. 4C). In general, Fig. 4 demonstrates a greater surface change in Predicta compared to Beautifil Kids. This can be attributed to a potentially higher release of ions in Predicta, which could impact the stability of its resin matrix, making it more susceptible to degradation. Our paper provides further support for this explanation through the microhardness results. Specifically, Predicta exhibited higher microhardness values after undergoing the pH-cycling challenge, primarily due to the significant release of ions from this material. However, it is important to mention that all changes in surface roughness remained below the critical value of 0.2 µm. Previous studies have indicated that restorations with a surface roughness exceeding this critical value are more prone to plaque accumulation and staining, which can lead to secondary caries [44, 45]. Therefore, the polishability of dental restorations is crucial for ensuring smooth surfaces that are less susceptible to topographic changes over time [44, 45].

Both color stability and reduced roughness values are important characteristics of dental restorations. Regardless of their quality and chemical stability, all dental restorations are subject to such changes. Thus, regular dental visits are necessary to monitor oral hygiene and inspect existing restorations for early signs of failure. In cases where color and surface changes are superficial, surface polishing is a non-invasive approach to prevent future complications such as bulk discoloration, stress concentration, and degradation [46, 47]. Additionally, early color changes at the tooth-restoration interface could indicate microleakage or early carious lesions, which can be managed when detected early [46, 47]. Restorative materials with ion-releasing capabilities may aid in preventing demineralization; however, it is important to recognize that the bioactivity of such materials may diminish over time. Developing rechargeable bioactive formulations could potentially ensure long-term bioactivity, as the resin matrix can replenish the lost ions released during the early lifespan of these restorations [14].

While this article provides insights into the performance of two bioactive restorative materials, future investigations should include testing these formulations using in vitro microbial models. Since these materials are already available in the market and approved for in vivo use, conducting randomized clinical trials would provide valuable data on their clinical performance and anti-caries activity. Additionally, evaluating other mechanical properties, such as fatigue and bonding strength, as well as the polymerization kinetics of these materials is recommended.

## Conclusion

In conclusion, this study demonstrates that bioactive restorative materials with ion-releasing capabilities exhibit excellent resistance to demineralization, particularly Predicta, which significantly increases the microhardness of the restored dentin. The color and surface roughness changes observed in the ion-releasing materials were comparable to those in the control group without bioactivity. These findings suggest the potential use of these bioactive formulations as an approach to avoid the occurrence of secondary caries in proximity to dental restorations.

## Data Availability

The data published in this paper is available upon request.
